# Rotatable central composite design versus artificial neural network for modeling biosorption of Cr^6+^ by the immobilized *Pseudomonas alcaliphila* NEWG-2

**DOI:** 10.1038/s41598-021-81348-8

**Published:** 2021-01-18

**Authors:** WesamEldin I. A. Saber, Noura El-Ahmady El-Naggar, Mohammed S. El-Hersh, Ayman Y. El-khateeb, Ashraf Elsayed, Noha M. Eldadamony, Abeer Abdulkhalek Ghoniem

**Affiliations:** 1 Microbial Activity Unit, Department of Microbiology, Soils, Water and Environment Research Institute, Agricultural Research Center (ID: 60019332), Giza, Egypt; 2 Department of Bioprocess Development, Genetic Engineering and Biotechnology Research Institute, City of Scientific Research and Technological Applications (SRTA-City), Alexandria, 21934 Egypt; 3 Department of Agricultural Chemistry, Faculty of Agriculture, Mansoura University, Mansoura, Egypt; 4 Botany Department, Faculty of Science, Mansoura University, Mansoura, Egypt; 5 Seed Pathology Department, Plant Pathology Institute, Agricultural Research Center, Giza, Egypt

**Keywords:** Microbiology, Environmental sciences, Environmental biotechnology

## Abstract

Heavy metals, including chromium, are associated with developed industrialization and technological processes, causing imbalanced ecosystems and severe health concerns. The current study is of supreme priority because there is no previous work that dealt with the modeling of the optimization of the biosorption process by the immobilized cells. The significant parameters (immobilized bacterial cells, contact time, and initial Cr^6+^ concentrations), affecting Cr^6+^ biosorption by immobilized *Pseudomonas alcaliphila*, was verified, using the Plackett–Burman matrix. For modeling the maximization of Cr^6+^ biosorption, a comparative approach was created between rotatable central composite design (RCCD) and artificial neural network (ANN) to choose the most fitted model that accurately predicts Cr^6+^ removal percent by immobilized cells. Experimental data of RCCD was employed to train a feed-forward multilayered perceptron ANN algorithm. The predictive competence of the ANN model was more precise than RCCD when forecasting the best appropriate wastewater treatment. After the biosorption, a new shiny large particle on the bead surface was noticed by the scanning electron microscopy, and an additional peak of Cr^6+^ was appeared by the energy dispersive X-ray analysis, confirming the role of the immobilized bacteria in the biosorption of Cr^6+^ ions.

## Introduction

Contamination of water by heavy metal ions is one of the major problems threatening the ecosystem^1,2^. Due to toxicity and tendency to be persistent in food chains, their occurrence causes a high-risk factor for human health and the environment^3^. Chromium is one of these metals, which is produced in the environment as a result of several industries, e.g. mines, surface finishing, fuel, energy production, pesticide, and steel and iron industries^4^. Although, it plays a functional role in the synthesis of nucleic acids, as well as, metabolism of proteins, fats, and carbohydrates, its toxicity arises from the oxidation status, which acting as the potential of mutagenic and carcinogenic to biological tissues in humans^5^.

The removal of such metals is an urgent issue from health, economic, and environmental points of view. Several conventional methods of heavy metals removal had been described earlier, such as ion exchange, coagulation, flotation, reverse osmosis, and electrochemical process^6^. The conventional methods were found to have many disadvantages, e.g. low efficiency especially in low concentration of metals, intensive energy requirement, and a secondary large quantity of impurities^7,8^. The alternative procedure is depending upon the biosorption process using some of the microorganisms, like fungi and/or bacteria, these techniques of biosorption use microorganisms in an immobilized, dead, or viable biomass form^9–11^.

Kinetics modes of the biosorption process had been proposed, e.g. transportation through the cell membrane, insertion into cell wall structure, precipitation, accumulation in the exopolymer layer of the cell, and/or through the oxidation–reduction process^2,12^. Importantly, the binding process of metals might be due to some polarized groups, e.g. phosphate, carboxyl, amino, amido, acetamido, sulfhydryl, and sulfate. The immobilization process is one of the protective techniques, which encapsulates the bacterial cells with the benefits of stabilizing cells, enhancing their viability against adverse environmental conditions, and confers additional protection during rehydration^13,14^.

The immobilization of microbial cells refers to the entrapment of cells without loss of vitality and functional activity, by which the matrix must be biocompatible and preserves the survival of cells and permeable to oxygen and also guarantee the influx of nutrients and the efflux of the toxic metabolites^15^. Immobilized cells had been also pronounced in several fields of the environmental, pharmaceutical, and food industry, for instance, immobilized cells of *Azotobacter nigricans* were efficient in Cu^2+^ removal^11^. Likewise, EL-Naggar et al.^16^ investigating the role of immobilized cells of *P*. *alcaliphila* in removing Cr^6+^ during Langmuir and Freundlich modeling.

Modeling by response surface methodology (RSM) has been extensively applied to assess the simultaneous influencing response factors and their interaction within the tested range, using a limited number of trials, Box–Behnken and central composite designs are two major sorts of RSM^11,16^. RSM is a worthwhile statistical mathematical system utilized for improving the experimental conditions through the discovery of the analytical relationship between inputs and outputs^17^. Therefore, modeling using RSM is recommended in the biotechnological process such as heavy metals removal^9,14^. Modeling by an artificial neural network (ANN) is another category, which is recently used to describe a wide range of processes, concerning their mathematical relationships. ANN can be, superly, replace the polynomial regression-based modeling approach, such as RSM, which modeling the complex nonlinear relationships. The ANN model is hypothetically more accurate because it includes all data points of an experiment^18,19^. The modeling procedure of ANN includes the choice of network architecture, establishing of the hidden layers and neurons number in each layer, learning, training, and, finally, validation and verification of the model^20^. Besides having advantages of the elucidation of the behavior of the biosorption process of metal along with wastewater management, ANNs have been, recently, applied successfully in various biotechnological fields as pattern recognition and forecasting^21,22^.

To the best of our knowledge, there have been no previous studies done on the modeling of the optimization of the biosorption process of Cr^6+^ by the immobilized cells of *P. alcaliphila*. The current study was carried out to maximize the biosorption process of Cr^6+^ by the immobilized cells of *P. alcaliphila* NEWG-2 as affected by the contact time and initial concentration of Cr^6+^ based on RSM and ANN.

## Materials and methods

### *P. alcaliphila* strain

In a previous study, *P. alcaliphila* NEWG-2 was found to be efficient in biosorption of Cr^6+^ and molecularly identified with an accession number of MN025267^16^. This strain was used during the current investigation. The bacterial strain was grown on slants contains a fermentation medium solidified with 15 g/L agar and incubated for 48 h at 25 ± 1 °C. *P. alcaliphila* NEWG-2 was regularly sub-cultured and preserved at 4 °C.

### Fermentation medium and bacterial enumeration

The fermentation medium for cell propagation was composed (g/l): MgSO_4_·7H_2_O (0.2), glucose (5), yeast extract (5), and pH 7.2. The medium was autoclaved at 121 °C for 20 min. In 250-mL Erlenmeyer flasks, *P. alcaliphila* NEWG-2 has grown on a 50 mL broth medium and incubated at 25 ± 1 °C for 48 h under shaking (100 rpm). After incubation period, the bacterial cells were separated by centrifugation at 5000 rpm for 20 min.

### Bacterial immobilization

Immobilization of *P. alcaliphila* NEWG-2 was performed in the sodium alginate gel according to the method of EL-Naggar et al.^23^. To make sphere beads, the cells of *P. alcaliphila* NEWG-2 has been mixed with sodium alginate gel, to count 10^5^ CFU per one ml of sodium alginate gel, with continual mixing at room temperature for 5 min. Using a 3-mL syringe, beads of inoculated sodium alginate (1.5 ± 0.2 mm in diameter) were created by dropping in a cold sterile CaCl_2_ (2.5%) with gentle mixing at room temperature. The beads were washed with distilled sterilized water several times to remove calcium chloride traces. For stabilization and rigidity, the beads were then held overnight in distilled sterilized water at 4 °C. Another set of beads were created using only sodium alginate without bacterial biomass.

The biosorption process was carried out in sterilized separating funnels, which were packed with the alginate beads without bacterial cells or alginate-bacterial beads. Solutions of Cr^6+^ with different concentrations were added and mixed with the beads and left at 30 °C, with initial pH 7. Samples from the various trials were collected from the separating funnel effluent and analyzed for the residual ions of Cr^6+^.

### Potassium dichromate (K_2_Cr_2_O_7_)

K_2_Cr_2_O_7_ (Sigma-Aldrich) was used to prepare various concentrations based on the content of Cr^6+^ ions. Samples of each sterilized concentration were allowed to contact alginate beads, inoculated or not, inside the separating funnel (Simax) for a specific time then fractions of the effluent samples were collected to determine the residual Cr^6+^.

### Screening Cr^6+^ removal factors using Plackett–Burman

Because the complete knowledge about the Cr^6+^ removal system is unavailable, the fractional-factorial Plackett–Burman matrix was performed to check the effect, contribution, and significance of the most important process parameters (bacterial cell immobilization in alginate beads, the contact time of alginate beads with Cr^6+^, and initial concentration of Cr^6+^) on Cr^6+^ removal by *P. alcaliphila*. The Plackett–Burman design is a screening matrix that aims to find out the importance of factor(s) and at which level in an experiment, consequently, unimportant (noise) factor(s) is screened out to avoid performing extensive study on relatively unimportant factors.

The experimental matrix was constructed from a mixture of categorical and numerical factors to validate the relative importance of the tested factors. The three independent variables were screened at low and high levels (Table 1). The design matrix and statistical calculations were operated using Minitab software (version 19, Minitab Inc., USA) (https://www.minitab.com/en-us/).

**Table 1:** Screening matrix of the Plackett–Burman design with the corresponding Cr^6+^ removal by *P. alcaliphila* NEWG-2.

Run	Screened parameter			Cr^6+^-removal, %	
	Bacteria (categorical factor)	Numerical factors			
		Time, h	Initial Cr^6+^ conc._,_ ppm	Response	Residual
1	Inoculated (+ 1)	180	450	64.02	0.59
2	Inoculated (+ 1)	480	150	70.09	2.36
3	Uninoculated (− 1)	480	450	33.45	0.54
4	Inoculated (+ 1)	180	450	67.97	4.55
5	Inoculated (+ 1)	480	150	65.42	− 2.31
6	Inoculated (+ 1)	480	450	46.08	− 3.56
7	Uninoculated (− 1)	480	450	33.02	0.11
8	Uninoculated (− 1)	180	450	44.48	− 2.21
9	Uninoculated (− 1)	180	150	66.98	2.20
10	Inoculated (+ 1)	180	150	79.88	− 1.63
11	Uninoculated (− 1)	480	150	53.86	2.87
12	Uninoculated (− 1)	180	150	61.28	− 3.50

### Modeling using RSM

The modeling technique of RSM was employed. For multiple regression analysis, a properly designed experiment was performed to simultaneously resolve multivariate equations. Two variables at five levels have been tested using a rotatable central composite design (RCCD). The tested independent variables selected for the modeling of the Cr^6+^ biosorption process using the immobilized *P. alcaliphila* NEWG-2 were; contact time between bacterial-alginate complex with Cr^6+^ and the initial Cr^6+^ concentration (Table 2).

**Table 2:** Experimental data of Cr^6+^ removal, based on the RCCD matrix of the independent factors and the corresponding predicted values obtained of the two tested models (RCCD and ANN).

Run	Independent variable		Cr^6+^ removal, %				
			Experimental	RCCD		ANN	
	Contact time, min	Initial Cr^6+^ conc., ppm		Predicted	Residual	Predicted	Residual
1^a^	60 (− 1)^b^	230 (− 1)	34.85	33.79	1.06	34.21	0.64
2	60 (− 1)	230 (− 1)	34.05	33.79	0.26	34.21	− 0.16
3^a^	300 (1)	230 (− 1)	75.12	77.41	− 2.29	73.72	1.40
4	300 (1)	230 (− 1)	74.12	77.41	− 3.29	73.72	0.40
5^a^	60 (− 1)	370 (1)	57.23	58.09	− 0.86	57.22	0.01
6	60 (− 1)	370 (1)	60.73	58.09	2.64	57.22	3.51
7^a^	300 (1)	370 (1)	74.34	76.94	− 2.60	72.71	1.63
8	300 (1)	370 (1)	74.44	76.94	− 2.50	72.71	1.73
9^a^	10.3 (− 1.414)	300 (0)	30.21	32.70	− 2.49	29.90	0.31
10	10.3 (− 1.414)	300 (0)	32.21	32.70	− 0.49	29.90	2.31
11^a^	349.7 (1.414)	300 (0)	81.11	76.88	4.23	80.77	0.34
12	349.7 (1.414)	300 (0)	79.41	76.88	2.53	80.77	− 1.36
13^a^	180 (0)	201 (− 1.414)	61.27	59.90	1.37	60.50	0.77
14	180 (0)	201 (− 1.414)	60.77	59.90	0.87	60.50	0.27
15^a^	180 (0)	399 (1.414)	76.53	76.75	− 0.22	78.39	− 1.86
16	180 (0)	399 (1.414)	78.53	76.75	1.78	78.39	0.14
17^a^	180 (0)	300 (0)	99.61	97.02	2.59	97.22	2.39
18	180 (0)	300 (0)	99.11	97.02	2.09	97.22	1.89
19	180 (0)	300 (0)	91.61	97.02	− 5.41	97.22	− 5.61
20	180 (0)	300 (0)	91.01	97.02	− 6.01	97.22	− 6.21
21	180 (0)	300 (0)	99.58	97.02	2.56	97.22	2.36
22	180 (0)	300 (0)	99.08	97.02	2.06	97.22	1.86
23	180 (0)	300 (0)	95.21	97.02	− 1.81	97.22	− 2.01
24	180 (0)	300 (0)	95.71	97.02	− 1.31	97.22	− 1.51
25	180 (0)	300 (0)	99.59	97.02	2.57	97.22	2.37
26	180 (0)	300 (0)	99.69	97.02	2.67	97.22	2.47

^a^Nine runs were randomly selected by the software for validating the model of ANN, the other 17 runs were used for training.

^b^The number between parentheses is the coded values of the tested parameters.

The efficiency of the biosorption process was evaluated by measuring the response variable (Cr^6+^ removal, %), which depends on the two input factors. The design matrix of RCCD, in terms of coded units, contained four factorials (± 1), four axial (± 1.414), and five center (0) points; the latter is to estimate the pure error. The relationship between actual values and the coded values of the tested parameters is calculated by the following equation:

$${x}_{i}=\left({X}_{i}- {X}_{0}\right)/{\Delta X}_{i}$$

where *x*_*i*_ is the coded value of an independent factor, *∆X*_*i*_ is the step-change in the actual value of the variable *i*, *X*_0_ is the actual value of an independent factor at the center point and *Xi* is the actual value of an independent factor.

The Cr^6+^ removal percent was determined as the response variable. The experimental data were statistically assessed by using multiple regression analysis, then F-test, values of correlation coefficient (R^2^), prediction error sum of squares (PRESS), predicted R^2^ and adjusted R^2^ were checked to compare and evaluate the significance of the regression model. The selected polynomial quadratic model was fitted to the next second-order equation:

$${\text{Y}} =\upbeta _{0} + \mathop \sum \limits_{{\text{i}}}\upbeta _{{\text{i}}} {\text{X}}_{{\text{i}}} + \mathop \sum \limits_{{{\text{ij}}}}\upbeta _{{{\text{ij}}}} {\text{X}}_{{\text{i}}} {\text{X}}_{{\text{j}}} + \mathop \sum \limits_{{{\text{ii}}}}\upbeta _{{{\text{ii}}}} {\text{X}}_{{\text{i}}}^{2}$$

where Y is the Cr^6+^ removal percent; β_ij_, is the interaction coefficients; β_ii_ is the quadratic coefficients; β_0_ model constant; β_i_, is linear coefficients; X_i_ and X_j_ are the independent factors.

The predicted response value was calculated based on the preceding equation model was subjected to laboratory validation to confirm the fitness and accuracy of the theoretically estimated value of each factor.

### Determination of residual Cr^6+^

Collected sample fractions were examined for the residual Cr^6+^. The residual Cr^6+^ concentrations were assessed using Atomic Absorption Spectrophotometer “Buck Scientific Accusys 211 series, USA by an air/acetylene flame system”^24^, then the Cr^6+^-removal percent was calculated.

### Modeling using ANN

A fully connected neural networks platform was constructed with one hidden layer, all nodes within the layer have the same activation function (Tan H sigmoid function, exp(− x^2^)). Response data of the RCCD matrix was used to train the machine and develop the predictive model. The data were portioned into three sets, i.e., training, testing, and cross-validation, in which 17 runs were used randomly for training, while the other 9 data sets were used for testing and validation.

**Figure 1:**
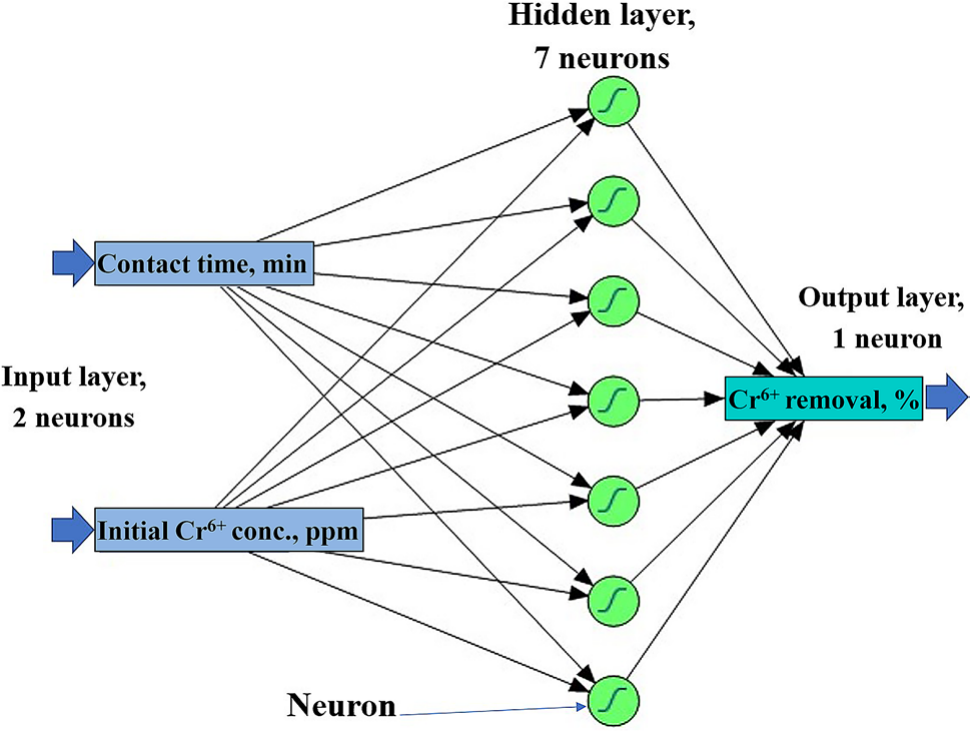
General architecture scheme of the artificial neural network containing one input layer (2 neurons), a hidden layer (7 neurons), and an output layer (one neuron).

The neural network had three layers. The ANN topology was designated as 2-h-1. The input layer composed of two neurons (contact time and initial Cr^6+^ concentration, ppm) and the output layer has one neuron (Cr^6+^ removal %), which is fixed by the number of the tested independent and response factor, respectively. The in-between layer was tested using h neurons that varied from 3 to 10 in a single hidden layer (Fig. 1).

The trial-and-error search method was applied to train the ANN until a minimum of the root mean square error (RMSE) and the sum of squared errors (SSE) was reached, accompanied by the highest value of the (R^2^) in the validation process. The trained network performance test was calculated based on the precision of the neural network to predict outputs that are either similar or very close to the response target value.

### Testing the fitness of RCCD and ANN models

To assess the fitness of Cr^6+^ biosorption models of RCCD and ANN models, R^2^, RMSE, mean absolute deviation (MAD), and SSE were used to compare models. Besides, the values predicted by both models were plotted against the corresponding trial values to explore the fitness of models.

### Trial design and statistical examination

Both the statistical regression analysis of the RCCD matrix with the experimental results and also the analysis of variance (ANOVA) were accomplished using the statistical software package Design-Expert (version 12, Stat-Ease, Minneapolis, USA) (https://www.statease.com/software/design-expert/). The ANN topology was set up using JMP 11 software (JMP, Version 11 SAS Institute Inc., Cary, NC, 1989–2019), which enables training, validating, and testing using experimental data with several hidden neurons. Training of ANN was performed using 17 randomly selected runs by the software, whereas the other 9 runs were used to check the validity of the trained ANN model. To enhance the prediction accuracy of both models, experiments of RCCD were repeated twice, each with three replicates. The mean of each experimental run was calculated.

### Surface morphology analysis

The immobilized *P. alcaliphila* NEWG-2 samples were coated with gold before and after the biosorption process of Cr^6+^ and examined by Scanning Electron Microscopy (SEM).

### Energy-dispersive X-ray analysis (EDX)

The dehydrated immobilized *P. alcaliphila* NEWG-2 samples were examined before and after the biosorption process of Cr^6+^ using TEM/EDX to determine the elemental sample composition.

## Results and discussion

### Screening of Cr^6+^ removal using Plackett–Burman

It is already known that Plackett–Burman is used to achieve two main important approaches; the first is to determine the significance of the examined factors and the second is to detect the level (high or low) that significant factor(s) should be tested around. The main goal, herein, was to determine the starting point of each of the involved variables in the biosorption of Cr^6+^.

A combination of one categorical element (bacterial inoculation) and two numerical elements (contact time of alginate beads with Cr^6+^ and initial concentration of Cr^6+^) were investigated, based on the matrix of Plackett–Burman design, to estimate their significance and relative importance on Cr^6+^ removal (Table 1). The results show obvious variation among the various runs, yet, the values of the residuals recorded remarkably lower values. Residuals are the differences between the experimental (observed) value of the dependent variable (Cr^6+^ removal) and its corresponding predicted value at each data point. The lower the value of the residuals, the fitness of the model data that, consequently, signifies the accuracy of the parameter selection.

The statistical analysis of the Plackett–Burman results was performed. Pareto chart (Supplementary Fig. 1) of the standardized effects figures the relative magnitude and the statistical significance of the three variables in the descending order. Parameters that pass the reference line (2.31) are significant at 0.05, therefore, the three tested parameters were significant on Cr^6+^ removal by the investigated bacterium.

According to Table 3, the impact of the three tested variables was explored at the probability (*P*) level of 0.05, in which model terms with *P* < 0.05 are significant. The three tested variables show significance (*P* < 0.05). Initial chromium concentration followed by contact time recorded the highest contribution percent with a negative effect, whereas the bacterial inoculation was positive in its effect. If the effect is negative, a lower concentration is to be required during further optimization studies, the vice versa. Also, the positive effect of bacterial inoculation reflects the importance of the tested bacteria in the bioremoval process under immobilization conditions.

**Table 3:** The effect, contribution, and corresponding *F*- and *P*-values for Cr^6+^ removal recovered by *P. alcaliphila* NEWG-2 using Plackett–Burman design.

Term	Effect	Contribution, %	*F* value	*P* value
Model		96.85	82.03	0.000
Linear		96.85	82.03	0.000
Bacteria	16.731 (+ 1)	34.02	86.44	0.000
Contact time	− 13.786	23.1	58.69	0.000
Initial chromium concentration	− 18.082	39.74	100.97	0.000
Error		3.15		
Lack-of-fit		1.73	1.22	0.427
Pure error		1.42		
Total		100		
Coefficient of determination (R^2^)	96.85			
Adjusted R^2^	95.67			
Predicted R^2^	92.92			

To evaluate the aptness of the data and select the model with the best fit, R^2^, and adjusted R^2^ are estimated. R^2^ defines the variation quantity in the experiential response values that are described by the factor(s). Adding items to the model lead to get bigger R^2^, but adjusted R^2^ is not, because it depends on the significance of the factors, not their number, of the model. However, the higher the adjusted R^2^ the additional accuracy of the relationship between the factors and the response (Cr^6+^ removal), consequently, the model fits well the data. Predicted R^2^ illustrates how well the model predicts for the responses in the new experiments, without over-fitting. Increased R^2^ predicted values suggest high prediction efficiency of the model. Current values show that all the kinds of R^2^ display high validity with the selected variables, being 96.85 (R^2^), 95.67 (adjusted R^2^), and 92.92% (predicted R^2^).

Free microbial cells have a key role in the biosorption of heavy metals. Various mechanisms were suggested, such as ion exchange, complexation, precipitation, chelation^25^. Likewise, the biosorption arises through interaction among charges of metal ions, exopolysaccharides, and cell surface. In this respect, several functional groups, i.e., amine, sulfhydryl, phosphate, phosphodiester, hydroxyl in polysaccharides, carboxyl groups in proteins provides the polymer an overall negative charge^26^. The biosorption by *Pseudomonas* genus could effectively occur through exopolysaccharides, which have a high content of uronic acid that enhanced their capability with binding to metal ions^27^.

Immobilization techniques have been reported to boost the biological reaction kinetics, especially reaction rate, in which the immobilization process stimulates the production of the exopolysaccharides without altering specific growth rates^28^. For example, the immobilized *Chryseomonas luteola* showed efficiency in the sorption of copper, nickel, cobalt, and cadmium compared to alginate beads alone^27^.

On the other side, the role of sodium alginate beads alone was stated and was found to absorb some of the heavy metals, e.g., Cr^6+^ ^16^ and Cu^2+^ ^11^ ions. The sodium alginate is a natural polymer produced by marine algae, with a chemical structure of mannuronate and guluronate arranged with 1, 4-linkage. The ionic strength, permeability, viscosity, and stability could be different due to molecular weight and the ratio of mannuronate to guluronate^29^. Briefly, all these characters may play a vital task in the sorption process of metals by sodium alginate beads.

### Modeling of Cr^6+^ removal by RCCD

Built on the previous screening of Plackett–Burman the three investigated factors, were subjected to further study, regarding their interaction and modeling process. Contact time and initial chromium concentration were tested around the low levels, because of their negative effect, while all experiments were performed using inoculated beads (alginate-immobilized *P*. *alcaliphila*), which showed a positive effect. The empirical design of RCCD of RSM was applied, and the modeling technique was accomplished based on the quantitative data acquired from the experimental design. The RCCD has two factors at five levels for optimizing the two process variables for maximum removal of Cr^6+^.

The results of Table 2 represent the experimental response values of Cr^6+^ removal obtained by the various RCCD combinations in 26 runs; also the predicted values of RCCD and their residuals were introduced. Residual is the difference between the experimental variable (Cr^6+^ removal) and its corresponding predicted value at each data point. Lower values of the residuals reflecting a close correlation between the experimental values and the RCCD-predicted values, subsequently, the appropriateness of the generated model.

### Multiple regression analysis

To select the most appropriate kind of regression, the linear, interactive, and quadratic models were compared using multiple regression analyses based on the experimental data (Table 4). Analysis of the sequential model sum of squares shows that the *P* value of the quadratic model is the only significant model (*P* < 0.0001), the lack-of-fit value displays insignificant behavior, which a pre-request for the model to be fitted. The other summary of the fit statistics confirms that the quadratic model is the most fitting one, having the largest values of predicted R^2^ (0.9779), adjusted R^2^ (0.9827), and R^2^ (0.9862) with a small standard deviation (3.005). Models with supreme adjusted R^2^ and the predicted R^2^ are desired^17^.

**Table 4:** Fitness and adequacy of the models based on the experimental data of the design matrix of the RCCD.

Source	Sum of square	*df*	Mean square	*F* value	*P* value
**The sequential model sum of squares**					
Linear versus mean	4470.55	2	2235.27	5.98	0.0081
2FI versus linear	306.53	1	306.53	0.81	0.3768
Quadratic versus 2FI	8105.67	2	4052.84	448.77	< 0.0001
Residual	132.87	18	7.38		
**Lack-of-fit**					
Linear	8474.31	6	1412.39	202.61	7.4780
2FI	8167.78	5	1633.56	234.33	0.0000
Quadratic	62.11	3	20.70	2.97	0.0612
Pure error	118.51	17	6.97		

Source	SD	R^2^	Adjusted R^2^	Predicted R^2^	PRESS
**Model summary statistics**					
Linear	19.329	0.3422	0.2850	0.1895	10,587.79
2FI	19.407	0.3657	0.2792	0.1506	11,096.32
Quadratic	3.005	0.9862	0.9827	0.9779	288.7478

*df* the degree of freedom, *2FI* two factors interaction, *SD* standard deviation, *R*^*2*^ determination coefficient, *PRESS* the sum of squares of prediction error.

Accordingly, the quadratic second-order polynomial equation was selected and modeled using the results of RCCD. The resultant prediction equation was applied for the given levels of contact time and initial Cr^6+^ concentration, and so, the equation structure, in terms of coded factors, is assumed to be:

$$\begin{aligned} {\text{Cr}}^{{{6} + }} \,{\text{removal}}, \, \% \, & = { 97}.0{2 } + {15}.{62 }\,\left( {{\text{contact}}\,{\text{time}}} \right) \, + {5}.{96 }\,({\text{initial}}\,{\text{Cr}}^{{{6} + }} \,{\text{conc}}.) \, \\ & \quad - {6}.{19}\,({\text{contact}}\,{\text{time }} \times {\text{ initial}}\,{\text{Cr}}^{{{6} + }} \,{\text{conc}}.) \, - {21}.{12 }\,\left( {{\text{contact}}\,{\text{time}}} \right)^{2} \\ & \quad - {14}.{346 }\,({\text{initial}}\,{\text{Cr}}^{{{6} + }} \,{\text{conc}}.)^{2} . \\ \end{aligned}$$

### ANOVA examination

To check if the equation adequately reflected a genuine association between the independent variables (contact time and initial Cr^6+^ conc.) and the response (Cr^6+^ removal), ANOVA was performed (Table 5).

**Table 5:** ANOVA of Cr^6+^ removal based on the experimental results of the RCCD matrix.

Source	Sum of square	*df*	Mean square	*F* value	*P* value
Model	12,882.75	5	2576.55	285.30	< 0.0001
Contact time, min	3902.95	1	3902.95	432.17	< 0.0001
Initial Cr^6+^ conc., ppm	567.60	1	567.60	62.85	< 0.0001
Contact time × initial Cr^6+^ conc	306.53	1	306.53	33.94	< 0.0001
(Contact time)^2^	6203.77	1	6203.77	686.94	< 0.0001
(Initial Cr^6+^)^2^	2863.51	1	2863.51	317.08	< 0.0001
Residual	180.62	20	9.03		
Lack-of-fit	62.11	3	20.70	2.97	0.0612
Pure error	118.51	17	6.97		
Total	13,063.37	25			
SD	3.005				
Coefficient of variation	3.996				
PRESS	288.748				
Adequate precision	44.554				
R^2^	0.9862				
Adjusted R^2^	0.9827				
Predicted R^2^	0.9779				

*df* the degree of freedom, *PRESS* the sum of squares of prediction error.

The high model *F*-value of 285.30 together with the low lack-of-fit *F*-value (2.97) means that the model is significant. Model fitting is required to be significant; on the other hand, the lack-of-fit is wanted to be insignificant because its significance is a source of trouble to the model. Both model and lack-of-fit are opposite to each other.

The calculation of the relative dispersion of the experimental data from the predictions of the polynomial model of the second-order indicates the coefficient of variance is sufficiently low (3.996%) to designate that the deviation between the experimental and prediction values at each data point is low^18^.

The value of R^2^ was calculated to be 0.9862, which denotes that 98.62% of investigational data were well-matched. The adjusted R^2^ value (0.9827) is also high to support the model significance. The predicted R^2^ of 0.9779 is in reasonable agreement with the adjusted R^2^ indicating the wellness of the model to predict new observations. Increased R^2^ values indicate that the model is stronger and it has higher predictive efficiency for the response^30^.

The sum of squares of prediction error (PRESS) value was reasonably low (288.748), reflecting the lower opportunity of an error through the experimental work, consequently, and the predicted values. Estimating the signal to noise ratio, using adequate precision, shows a ratio of 44.55, the present ratio is high enough to indicate an adequate signal, a ratio greater than 4 is desired which indicate that the tested model can precisely be used to navigate the space of design^18^.

The two independent variables were further statistically examined for interaction and quadratic effect on Cr^6+^ removal. All *P* values were found to be < 0.0001, values less than 0.05 denote that model terms are significant. In the present study, all model terms are significant^31^. All the adequacy and fitness tests confirm the model's effectiveness in predicting the specific model fits at each point of the design space.

### Residuals normality and 3D-surface

**Figure 2:**
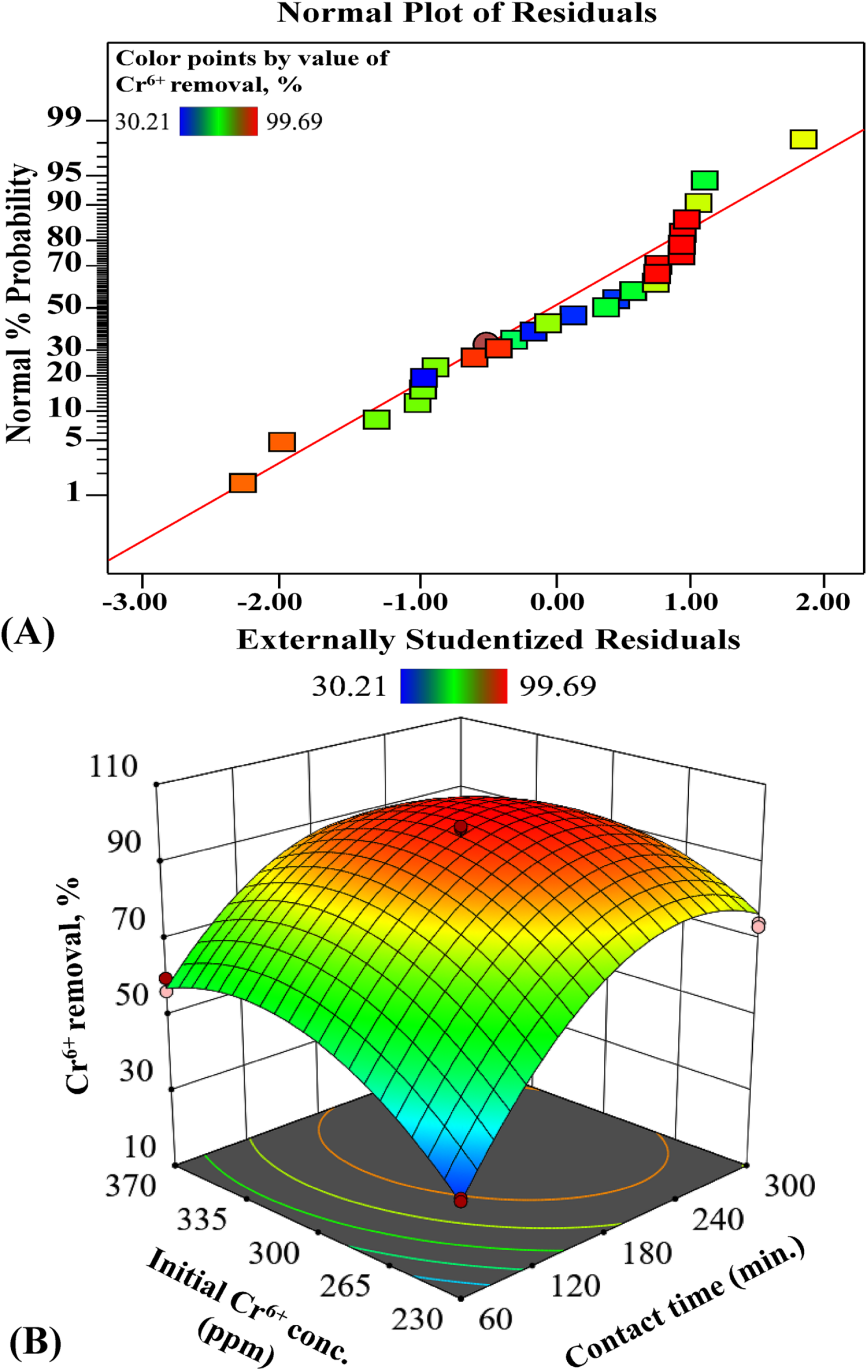
Normal probability plot of externally standardized residuals (**A**) and three-dimensional surface curve of Cr^6+^-removal as combined influence of contact time and initial Cr^6+^ conc. by RCCD (**B**). This figure was created by using Design Expert version 12 for Windows software.

To test the normality of the residuals, the normal probability plot of the residuals is displayed in Fig. 2A. Expect some scatter, there are no definite patterns, like curves, could be observed. On the other hand, the residual points were tightly clustered along the standard line indicating that the residuals must follow the normal distribution^32^. The 3D response surface plot in relation to the two factors (Fig. 2B) was used to understand both the linear and the interaction effects of the two tested variables, also to calculate the optimal level of each factor for maximum removal of Cr^6+^ ions.

The elliptical curve of the response surface plot displays a clear interaction between the tested parameters^20^. However, the 3D plot displays that Cr^6+^ removal reached its peak (99.995%) with the increment of initial Cr^6+^ conc. around nearly the center points of the design, reflecting the precision of the selected range of both tested factors.

### Experimental validation

To resolve the optimal combination of the tested variables, which maximize the response. The optimal predicted levels of both tested factors were estimated using the modeling regression equation and were found to be 224.6 min of contact time between chromium and immobilized bacteria and 315.98 ppm of initial Cr^6+^ concentration, at these conditions, Cr^6+^ removal reached 99.995%. These theoretical estimations from the equation were experimentally validated and the response was found to be 99.81%. This value is closely related to the theoretical value, substantiating the suitability of the developed model.

### Modeling of Cr^6+^ removal by ANN

The most common and popular multilayer feed-forward ANN architecture with the sigmoidal function was developed for modeling the bio-removal process of Cr^6+^ by the immobilized *P*. *alcaliphila*. The RCCD matrix and their respective experimental response were used for training the ANN. The network has two input nodes (contact time and initial Cr^6+^ concentration) and one output node (Cr^6+^ removal).

To determine the architectural structure and the best number of neurons in the hidden layer, numerous hidden neurons, and various combinations of ANN-specific parameters such as learning rate, as well as, the initial weight and bias value of each layer were tested. Hence, the optimal number of neurons in the hidden layer of the ANN was found to be 7 (Fig. 1). Consequently, the resulted architecture of ANN that has the maximum performance was found to be 2–7(h)-1. Previous work found that the optimal number of neurons in ANN architecture for modeling biosorption of chromium was 4–10-1^22^.

The generality of the ANN model was certified by minimizing the errors in training and validation. The network was trained until the R^2^ reached its maximum and RMSE, MAD and SSE recorded the lowest values (Table 6). Hence, the predicted values, for each experimental run based on the developed ANN model, were estimated and given along with the predicted RCCD and experimental values in Table 2. Because any linear regression model is not always appropriate for the data, residuals are generally used to assess the aptness of the model by defining residual values and determining its trend. The residuals presented as the variance between the experimental value of Cr^6+^ removal and its corresponding predicted data point at each dataset was found to be very low at all tested points. This implies that the ANN can fit the actual experimental data exactly. Recently, the ANN modeling has found its way in biosorption of toxic metals such as lead^21^, copper^28^, and zinc^33^, but none of them used immobilized cells during modeling process neither by RCCD nor ANN.

**Table 6:** Modeling comparison statistics of RCCD and ANN.

Measure	RCCD	ANN	
		Training	Validation
Coefficient of determination (R^2^)	0.9862	0.9872	0.9935
Root of mean square error (RMSE)	3.005	2.568	1.712
Mean absolute deviation (MAD)	17.892	1.834	1.594
Sum of squares due to error (SSE)	118.51	112.12	26.39
Number of runs used	26	17	9

### RCCD versus ANN models

Both RCCD and the well-learned ANN models were compared regarding their predictive capability to remove Cr^6+^ by the immobilized *P*. *alcaliphila* NEWG-2 strain. The statistical parameters that measure and compare the accuracy of both models were estimated (Table 6). The modeling ability of a given model is reliant on the high value of R^2^ and lower values of the RMSE, MAD, and SSE.

R^2^ measures the correlation between the response values and the predicted values, so, higher value (up to 1) reflects a strong correlation between both datasets. Commonly, RMSE is used in regression analysis to authenticate experimental results, since the lower value means that the data are concentrated around the line of best fit (prediction errors). MAD, is another statistic that determines the average dispersion of the data around the mean, a lower value indicates a lower spread of the data around the mean. Finally, SSE, another assessment of the goodness-of-fit, determines the total deviation of the response values from their fitted values, lower value implies more fitness of the model.

Given the preceding statistics, both models exhibited high predictive ability. However, comparing the two models reveals that RCCD is lower in R^2^ and higher in the other goodness-of-fit statistic than the ANN model. Therefore, the ANN model has a higher predictive ability than the RCCD model for Cr^6+^ removal by the immobilized *P*. *alcaliphila* NEWG-2. The current conclusion is conceding with that obtained by Shafi et al.^19^, who found that the ANN models were superior to RSM, recording lower RMSE, MAD, and chi-square and higher for R^2^.

**Figure 3:**
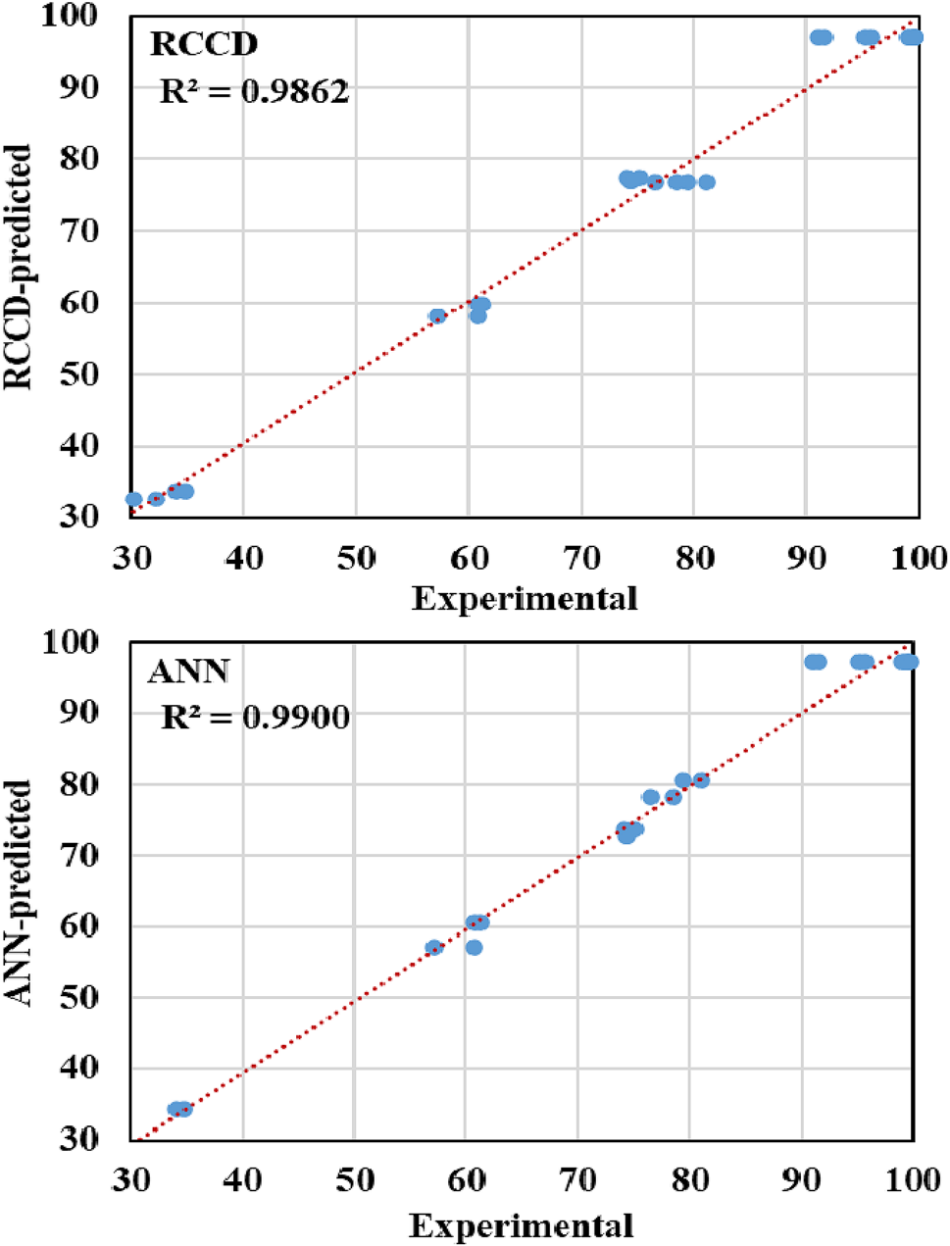
Actual versus RCCD and ANN predicted values for chromium removal by *P. alcaliphila* NEWG-2.

Likewise, another overall comparison was performed, in which the linear regression analysis between the actual values and those of predicted were drawn for both RCCD and ANN (Fig. 3). Again, the chart plot of ANN displays better fitting with a higher R^2^ value, which also infers that ANN gives improved optimization results compared to RCCD. The graph also shows that the ANN model prediction points lie much closer to the line of perfect prediction than the RCCD model. Thus, the ANN model has a significantly higher generalization capacity than the RCCD model.

However, there are some merits when modeling using RCCD i.e., the structured nature of the RCCD can demonstrate the contributions of each factor in the regression models, thus recognizing the insignificant factors, wither single, interaction, or quadratic, in the model and thereby can be eliminated from the model. Moreover, compared to RCCD, ANN modeling consumed extended computational time through many iterative calculations. However, the generated model by ANN had high predictive accuracy than RCCD. This can be attributed to its universal ability to approximate the nonlinearity of the system, compared with the restricted nature of RCCD to the second-order polynomial, which requires only a sole step calculation for a response surface model^18,20^. However, ANN has consistently performed better than the RCCD in all aspects.

The ANN models were adopted in various wastewater treatment, since these techniques are useful in forecasting effluent quality and estimation of metals in a given source, as well as, updating the prediction points upon selection of any input or output variables^33,34^. Contrarily, the other mathematical methods have difficulty in the prediction of desired output in wasted effluents, due to different types of metals, variety of salts, the variation of pH and temperatures^33^, wherein, the ANN models have been a promising technique in biosorption process of heavy metals in the industrial effluent.

### Surface morphology analysis

**Figure 4:**
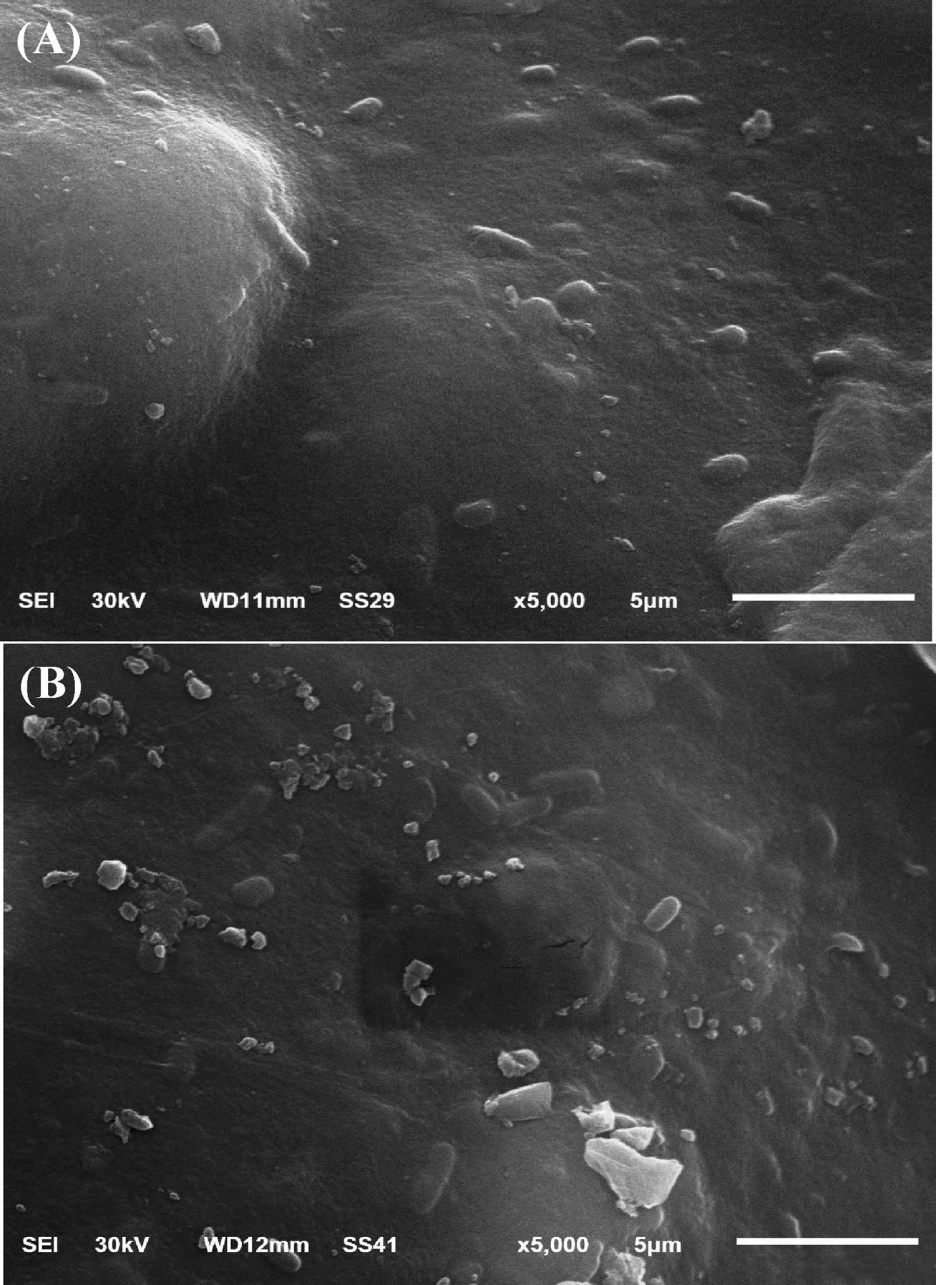
SEM micrograph of the immobilized *P. alcaliphila* NEWG-2: (**A**) before and (**B**) after biosorption of Cr^6+^.

The surface morphology of the immobilized *P. alcaliphila* NEWG-2 was examined by Scanning Electron Microscopy (SEM) before and after the Cr^6+^ biosorption. Figure 4A revealed a regular surface of the immobilized cells of *P. alcaliphila* NEWG-2 before the biosorption process. Figure 4B clearly shows the presence of glossy particles on the surface of the alginate beads after Cr^6+^ biosorption which are absent before Cr^6+^ biosorption.

### Energy-dispersive X-ray analysis (EDX)

**Figure 5:**
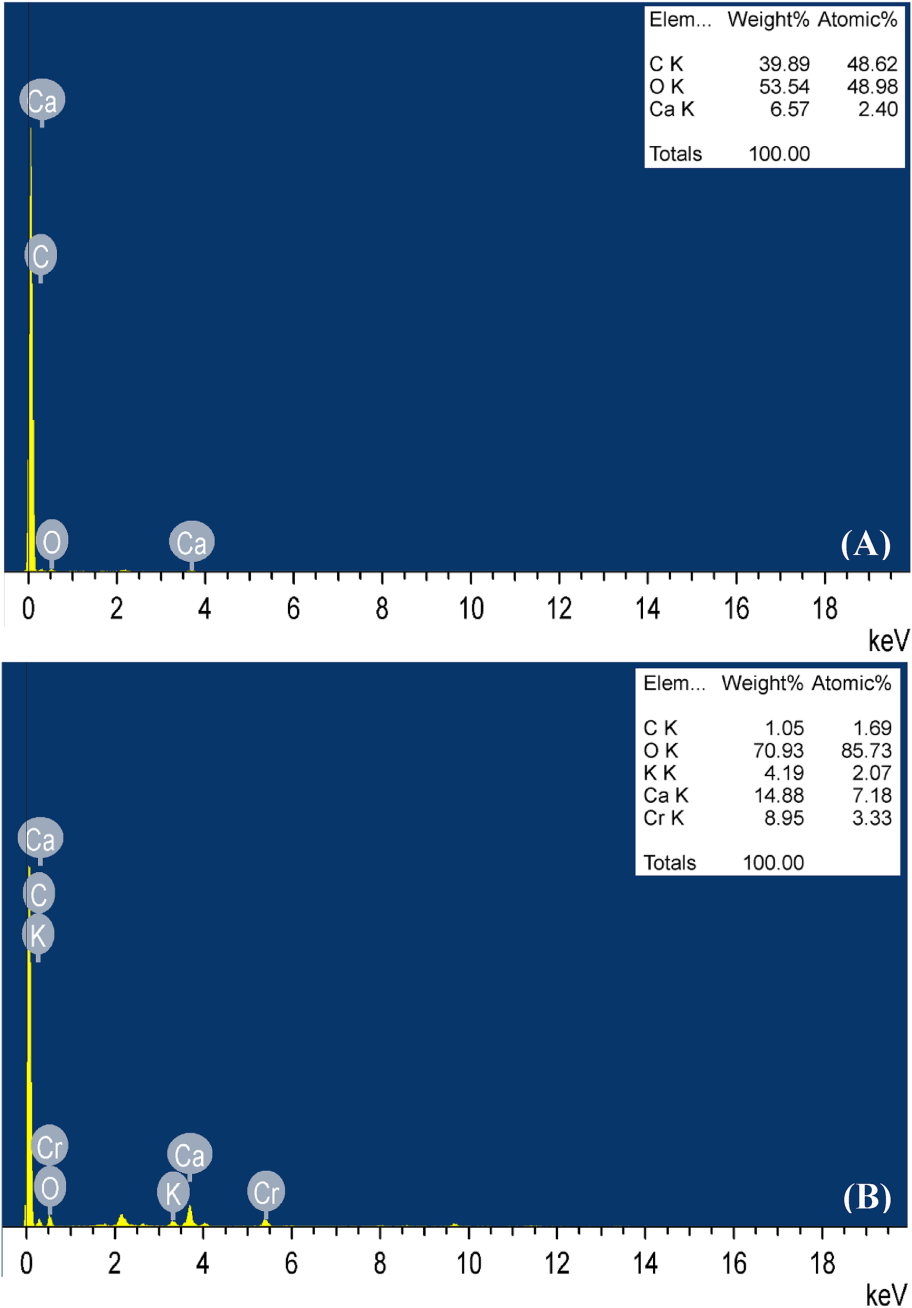
EDX analysis of the immobilized *P. alcaliphila* NEWG-2: (**A**) before and (**B**) after biosorption of Cr^6+^.

EDX was performed to determine the elemental composition and to verify the presence of Cr^6+^ attached to the immobilized *P. alcaliphila* NEWG-2 surface. Comparing the EDX spectrum before (Fig. 5A) and after (Fig. 5B) the Cr^6+^ biosorption process shows the presence of an additional Cr^6+^ peak after the Cr^6+^ biosorption compared to the EDX spectrum before the Cr^6+^ biosorption. That, in turn, proves the capacity of the immobilized *P. alcaliphila* NEWG-2 to remove Cr^6+^ from aqueous solutions. Regarding the overall biosorption process, there are two main topics. The first belongs to sodium alginate beads that were reported to absorb Cr^6+^ ions^16^, and the second belongs to the microbial cell.

### Different mechanisms of bacterial biosorption

Among microbial groups, bacteria have been identified to be one of the most important bio-sorbents. The biosorption capacity depends not only on the type of metal ions but also the type of bacteria, especially the cell wall that contains a variety of surface organic functional-groups, with a high affinity to binding metals^35^.

**Figure 6:**
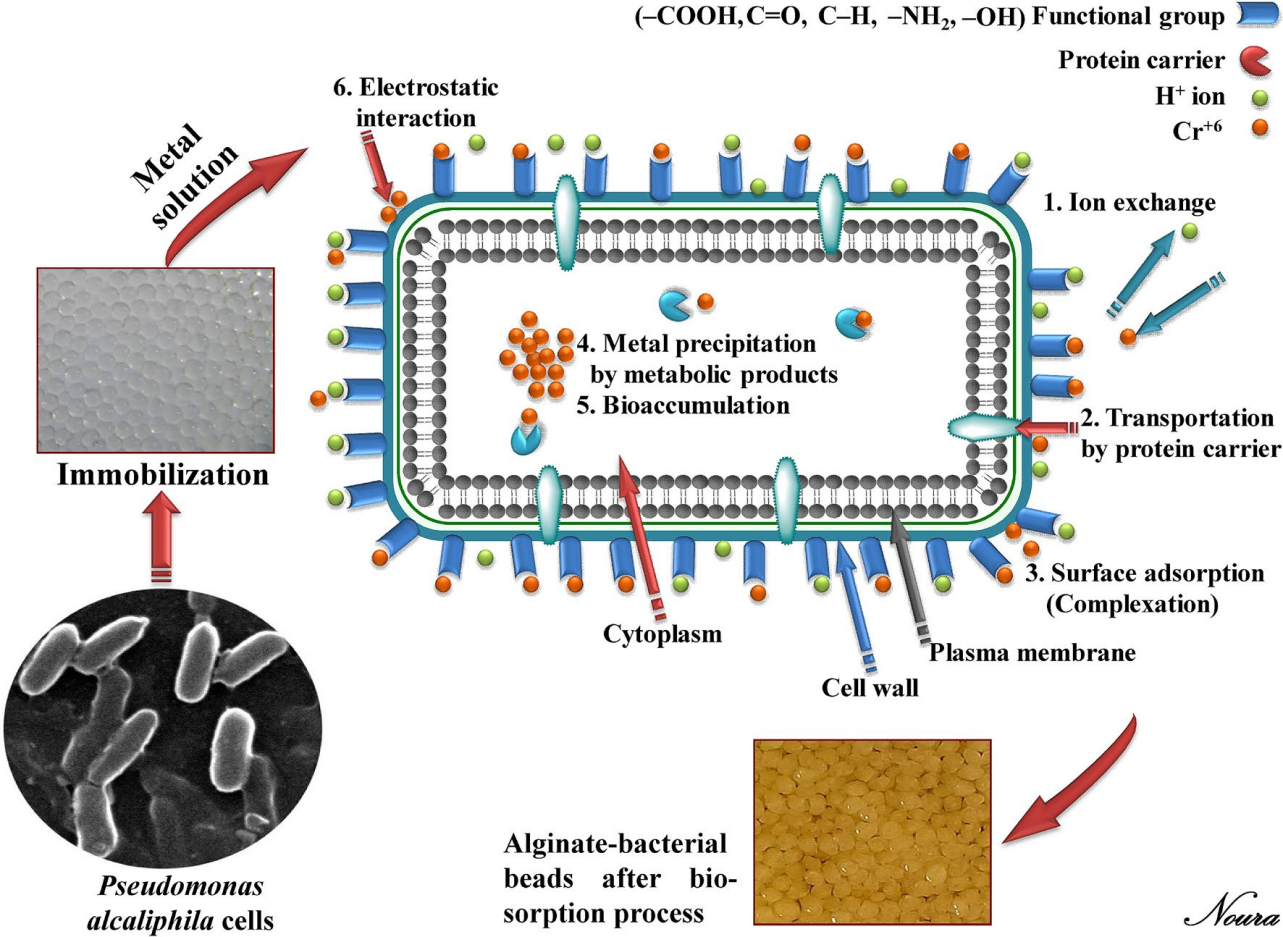
Schematic diagram showing the possible kinetic mechanisms of metal ions biosorption by the bacterial cell. The photograph was taken by using scanning electron microscope for *P. alcaliphila* NEWG-2 cells.

The kinetic biosorption process by individual bacterium biomass may have several mechanisms that work complementary or individually. The different mechanisms of biosorption are shown in Fig. 6. Generally, the microbial activity towards metal removal including two phases; physical adsorption or ion exchange at the cell surface, followed by a slower phase involving active metabolism-dependent transport of metal into bacterial cells^36,37^. It is, therefore, possible that the metal ion is transferred into the cell and reacts to form a precipitate and remaining within the cells, or forming an affine colloidal entrapped by extracellular polymers^38^.

In details, bacteria share several mechanisms for heavy metal biosorption, including ion exchange, physical adsorption on specific binding sites (negatively-charged functional groups) of the cell wall, complexation, diffusion, intracellular accumulation, or physicochemical interactions between the metal ions and the bacterial cell wall^39^.

Concerning *Pseudomonas* spp., the kinetic process of chromium biosorption suggests an efficient intracellular mechanism of chromate uptake by *P. aeruginosa* and the adsorption process has an endothermic nature^40^. The rhamnolipids (a low-molecular-weight biosurfactant) content of *Pseudomonas* spp. plays another important role in the bioremoval of chromium and its efficiency may be back to the anionic nature and complexation ability, therefore the bio-removal process is positively correlated with rhamnolipids quantity^36,37^. additionally, rhamnolipid production increased when *Pseudomonas* sp. was exposed to chromium^37^.

*P. aeruginosa* ASU 6a is an example of a bacterium that contains negatively-charged functional groups (carboxylate, phosphate, sulfhydryl, and amino groups) that were reported to play a vital role in the biosorption process^41^. However, ionic exchange by some of the functional groups (e.g., ‒NH, ‒OH, ‒CH, and ‒CONH) and electrostatic interaction were reported as the dominant mechanisms presented in biosorption of heavy metals by *P. plecoglossicida*^42^. Another work reported that the cell wall of *P. aeruginosa* S22 contains potential complexation sites such as carboxylate, phosphate, sulfahydryle, and amino (aspartic acid, glutamic acid, histidine, and cysteine) groups, the latter group, in especial, has a strong affinity for metal ions^43^.

## Conclusion

The Plackett–Burman design studies the significance of immobilized cell, contact time, and initial Cr^6+^ concentration on biosorption process by *P*. *alcaliphila* NEWG-2. Both RCCD and ANN models have a high accuracy in the modeling of the Cr^6+^ removal process. However, the ANN model showed to be more robust and accurate in assessing the prediction of dependent variables, with skill in prediction and generalization, within the training region, than the RCCD model. Finally, the ANN model could be recommended to be a fit technique in forecasting and accurate for heavy metals removal throughout industrial effluent treatment.

## Supplementary Information

Supplementary Figure S1.
