# Supplementary material for: Rotatable central composite design versus artificial neural network for modeling biosorption of Cr6+ by the immobilized Pseudomonas alcaliphila NEWG-2
Source: Sci Rep. 2021 Jan 18;11:1717. doi: 10.1038/s41598-021-81348-8 (PMC7814044; doi:10.1038/s41598-021-81348-8)
Supplement: Supplementary file 1 — Supplementary Figure S1. [file 41598_2021_81348_MOESM1_ESM.docx]

**Rotatable central composite design vs artificial neural network for** **modeling** **biosorption of Cr**^6+^ **by the** **immobilized** ***Pseudomonas alcaliphila* NEWG-2**

**WesamEldin I.A. Saber^1^, Noura El-Ahmady El-Naggar^2^, Mohammed S. El-Hersh^1^, Ayman Y. El-khateeb^3^,** **Ashraf Elsayed^4^, Noha M. Eldadamony^5^, Abeer Abdulkhalek** **Ghoniem^1^**

^1^Microbial Activity Unit, Department of Microbiology, Soils, Water and Environment Research Institute, Agricultural Research Center (ID: 60019332), Giza, Egypt.

^2^Department of Bioprocess Development, Genetic Engineering and Biotechnology Research Institute, City of Scientific Research and Technological Applications (SRTA-City), Alexandria 21934, Egypt.

^3^Department of Agricultural Chemistry, Faculty of Agriculture, Mansoura University, Egypt.

^4^Botany department, Faculty of Science, Mansoura University, Mansoura, Egypt

^5^Seed Pathology Department, Plant Pathology Institute, Agricultural Research Center, Giza, Egypt

**Corresponding Author’s information**

**Prof. Noura El-Ahmady Ali El-Naggar**

**Address:**

Bioprocess Development Department,

Genetic Engineering and Biotechnology Research Institute,

City of Scientific Research and Technological Applications,

New Borg El- Arab City, 21934, Alexandria, Egypt

**Tel:** (002)01003738444

**Fax:** (002)03 4593423

**E-mail:** nouraalahmady@yahoo.com


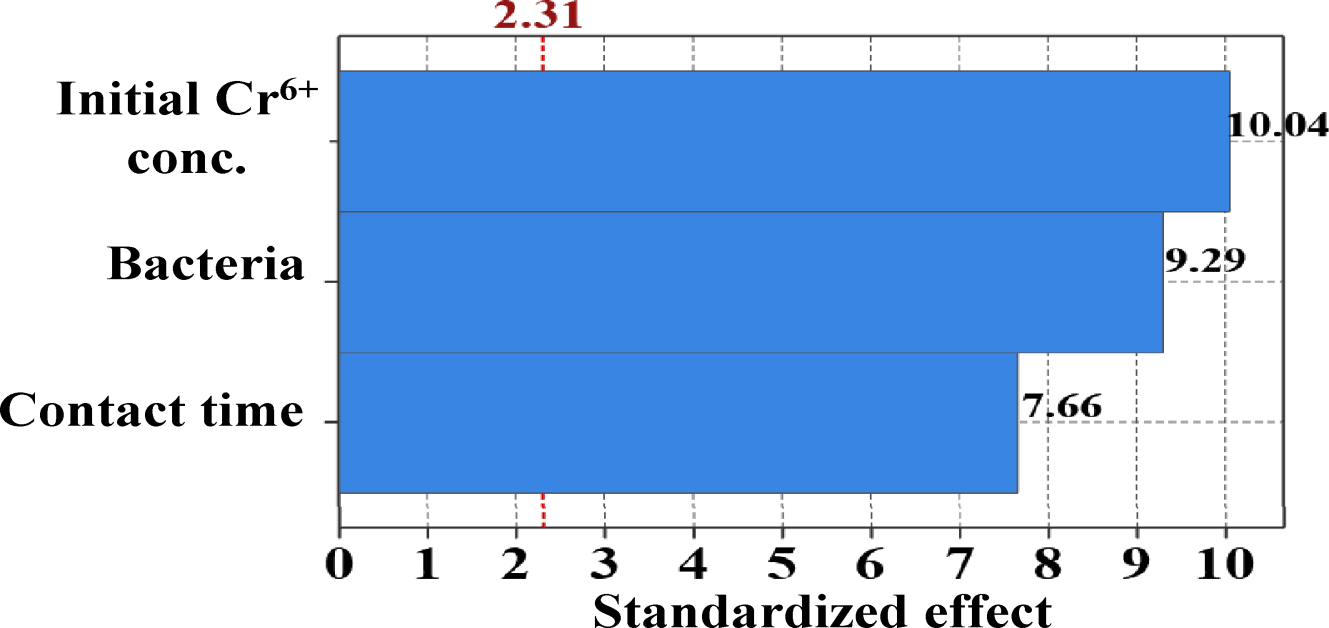


**Supplementary Figure 1.** Pareto chart, displaying the standardized effects of each of the three tested parameters
